# An efficient algorithm to identify the optimal one-bit perturbation based on the basin-of-state size of Boolean networks

**DOI:** 10.1038/srep26247

**Published:** 2016-05-19

**Authors:** Mingxiao Hu, Liangzhong Shen, Xiangzhen Zan, Xuequn Shang, Wenbin Liu

**Affiliations:** 1 Department of Physics and Electronic information engineering, Wenzhou University Wenzhou 325035, Zhejiang, China; 2 City College, Wenzhou University, Wenzhou 325035, Zhejiang, China; 3 School of Computer Science and Technology, Northwestern Polytechnic University, Xi'an 710072, China

## Abstract

Boolean networks are widely used to model gene regulatory networks and to design therapeutic intervention strategies to affect the long-term behavior of systems. In this paper, we investigate the less-studied one-bit perturbation, which falls under the category of structural intervention. Previous works focused on finding the optimal one-bit perturbation to maximally alter the steady-state distribution (SSD) of undesirable states through matrix perturbation theory. However, the application of the SSD is limited to Boolean networks with about ten genes. In 2007, Xiao *et al.* proposed to search the optimal one-bit perturbation by altering the sizes of the basin of attractions (BOAs). However, their algorithm requires close observation of the state-transition diagram. In this paper, we propose an algorithm that efficiently determines the BOA size after a perturbation. Our idea is that, if we construct the basin of states for all states, then the size of the BOA of perturbed networks can be obtained just by updating the paths of the states whose transitions have been affected. Results from both synthetic and real biological networks show that the proposed algorithm performs better than the exhaustive SSD-based algorithm and can be applied to networks with about 25 genes.

From a translational perspective, modeling gene regulatory networks provides a mathematical basis for system-based optimal therapeutic strategies. Boolean networks (BNs) and the more general class of probabilistic Boolean networks are one of the most popular approaches for modeling gene networks. In Boolean models, gene expression is quantized into only two levels: on or off, and the expression level of each gene is functionally determined by the expression states of some other genes using logical rules. The formalism of Boolean networks emphasizes the fundamental principles rather than quantitative biochemical details. It provides a nature framework to capture the dynamics of regulatory networks and the regulation of cellular states. From any initial state, the network will eventually settle down to one of a limited set of stable states, which are called *attractors*. The states that flow into the same attractor state make up a basin of attraction (BOA) of that attractor. The long-term behavior of BNs can be characterized by the size of all the BOAs. Generally speaking, attractors with larger BOAs tend to be more stable. Probabilistic Boolean networks (PBNs) extend the classical Boolean networks by introducing uncertainty in the rule structure. This uncertainty is motivated by randomness in the inference procedure, inherent biological randomness, and model stochasticity owing to latent variables^1^. PBNs are actually a family of constituent BNs that reflect the different contexts of the system. At each time, only one constitute BN is selected to determine the running of the network. Additionally, each gene is allowed to flip its value with a positive probability to reflect the perturbations of gene state caused by various environmental factors. This perturbation probability makes the Markov chain of a PBN irreducible and ergodic. Therefore, the long-term dynamical behavior can be described by a steady-state distribution (SSD). Those models have been used to study a number of biomolecular systems, such as the yeast-cell cycle, mammalian-cell cycle, Drosophila segment polarity network, regulatory networks of E. coli metabolism, and Arabidopsis flower morphogenesis^2^. It has been argued that the long-term behavior of such dynamic networks determines the phenotype or state of cell development, such as cell proliferation and apoptosis^3^.

The ultimate objective of gene-regulatory-network modeling and analysis is to design effective intervention strategies so that the dynamics of the network evolves toward desirable cellular states. Following the two seminal papers by Shmulevich in 2002^4^,^5^, the control of gene regulatory networks has been intensively studied within the framework of PBNs. Current control strategies can be classified into two categories: external control and structural intervention. External control “persuades” the system to move toward desirable states by flipping (or not flipping) the value of a control gene or genes over time. In biology, an example of external control is the activation of the well-known tumor-suppressor gene p53 in response to radiation which can rapidly inhibit cell growth and lead to apoptosis in a few hours^5^. The optimal control policies aim to maximally increase the steady-state distribution of desirable states and decrease that of undesirable states. Solving the optimal control problem requires applying a dynamic programming algorithm. However, it quickly becomes computationally infeasible as network size increases because the size of the search space for this optimization problem is *O*(2^*n*^). Several approximate and greedy algorithms have been proposed to find suboptimal solutions, such as the mean-first-passage-time control policy, the BOA control policy, the SSD control policy, and the conservative SSD control policy^6^. All these policies aim to reduce the risk of entering undesirable states that correspond to aberrant phenotypes of the modeled cells by some heuristic criterion. Recently, Yousefi *et al.* proposed to solve the optimal control problem by a linear-programming approach. Depending on whether desirable states are constrained, they presented the unconstrained optimal-intervention policy and the phenotypically constrained optimal-intervention policy, which can lead to the maximal phenotype alteration^7^.

Structural intervention directly changes the underlying network structure (wiring) to alter the long-term behavior (steady state) of the network^8^. In biology, this can be accomplished by introducing a transcription factor or drug that may affect the state of the target genes in some specific situations. For example, in developing countries, estrogen is often taken by women after menopause to slow down the aging process. However, the estrogen dose is critical because an overdose may increase the risk of developing breast or ovarian cancer. The simplest structural-intervention strategy is one-bit perturbation, which alters one output bit of the Boolean function for a specific gene. After a function perturbation, the original state transition matrix and SSD are both changed. The optimal structural intervention is to determine which perturbation on the truth table governing a BNp (where BNP is a Boolean network with perturbation *p*) or PBN would result in the maximal long-term effect on the dynamic behavior of the network. In 2007, Xiao *et al.* first studied how perturbation functions impact the BOA of Boolean networks^9^. In 2008 and 2012, Qian *et al.* adapted perturbation theory in finite Markov chains to derive an analytic solution to compute the shifted mass after a function perturbation^10^,^11^. In 2011, Bouaynayal formulated optimal intervention as an inverse-perturbation problem that can be solved by using standard convex optimization methods^12^. Although these approaches can find the optimal one-bit perturbation, their application is still limited to networks with about 10 genes. For example, the method in ref. 12 can only deal with BNs consisting of 10 to 15 genes, even when applying the current-best semi-definite program solvers. The main obstacle for methods based on matrix perturbation theory is that operations on the probability transition matrix (2^n^ × 2^n^) are too time consuming. Structural intervention can permanently change the SSD of the network because the fundamental functions have been changed. However, external intervention changes the SSD only when the control policies are performed. From this point of view, structural intervention has a greater potential impact on the dynamic behavior of networks than external intervention, and gene therapies are more likely to be developed by applying drug or genetic manipulation to alter extant cell behavior.

The probability mass of the SSD of BNs is mainly occupied by their attractor states. To avoid matrix operations, we can directly find the one-bit perturbation that maximally increases the BOA of desirable attractors and reduces the BOA of undesirable attractors. Xiao *et al.* proposed several algorithms to determine the optimal perturbation function based on the change in the size of the BOA^9^. However, their algorithms are very cumbersome and require closely observing the state transition changes before and after a perturbation. In this paper, we propose an algorithm that quickly determines the size of the BOA after a one-bit perturbation based on the basin of states (BOS) for each state.

The rest of this paper is organized as follows: The definitions, problem setting, and algorithm description are given in Section 2. The experimental results and discussion on both synthetic networks and real biological networks appear in Section 3. Finally, concluding remarks are given in Section 4.

## Methods

### Boolean networks and Probabilistic Boolean Networks

A Boolean network *G*(*V*, *F*) is defined by a set of nodes *V* = {*x*_1_, …, *x*_*n*_}, *x*_*i*_ ∈ {0, 1}, and a set of Boolean functions *F* = {*f*_1_, …, *f*_*n*_}, *f*_*i*_: 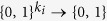. Each node *x*_*i*_ represents the expression state of a gene, where *x*_*i*_ = 0 means that the gene is off and *x*_*i*_ = 1 means that it is on. To update the node value, each node *x*_*i*_ is assigned a Boolean function 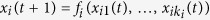 with *k*_*i*_ specific input nodes. Under the synchronous-updating scheme, all genes are updated simultaneously according to their corresponding update functions. The network’s state at time *t* is denoted by a binary vector **x**(*t*) = (*x*_1_(*t*), …, *x*_*n*_(*t*)). In the absence of noise, the state of the system at the next time step is


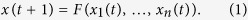


**Figure 1:**
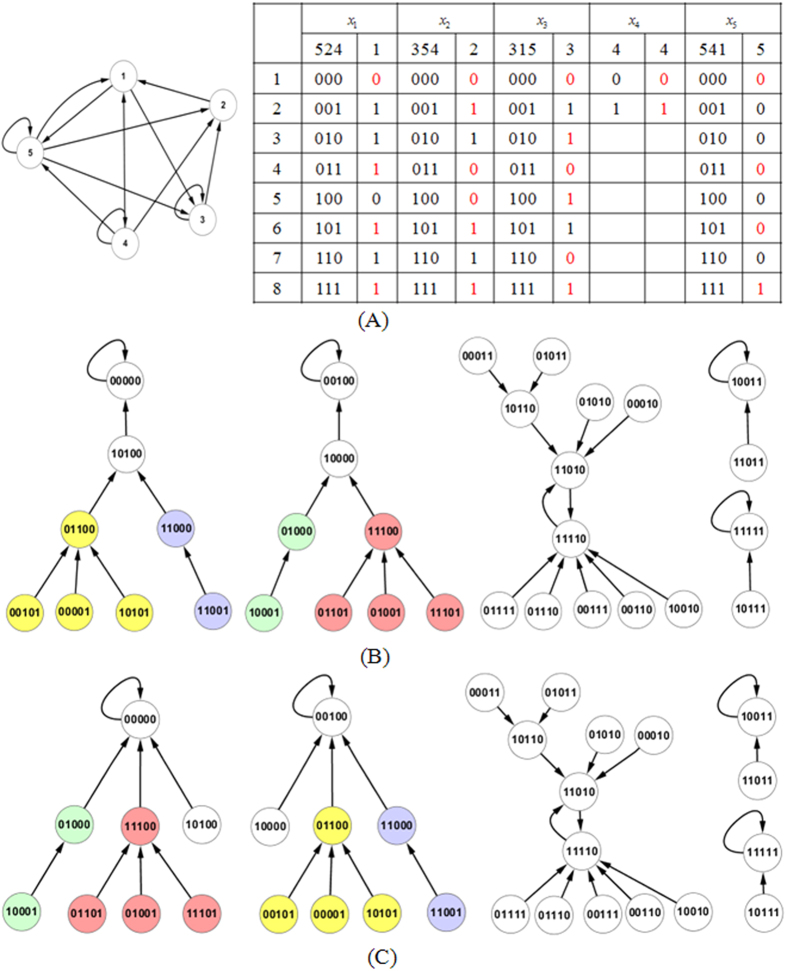
A simple BN consists of five genes with the maximum indegree 3. (**A**) The regulation relationships of genes and their corresponding truth table. The bits whose perturbation will destroy the current attractors are marked by red color in the truth table. (**B**) The state-transition diagram before any perturbation. (**C**) The state-transition diagram after perturbing 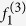.

In Boolean networks, there are generally two kinds of attractors: singleton attractors and cyclic attractors. The former consists of only one stable state while the latter consists of multiple stable states. Figure 1(A) shows a BN consisting of five genes and the corresponding truth table of all genes. Figure 1(B) shows the state transition diagram of this network. There four singleton attractors 00000, 00100, 10011, and 11111 and one cyclic attractor composed of two states 11010 and 11110. The sizes of their corresponding BOAs are 8, 8, 2, 2, and 12, respectively.

A PBN is composed of a family 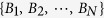 of BNs with a selection probability *c*_*i*_(1 ≤ *i* ≤ *N*). At each time, there is a switching probability to determine whether to select a constitute BN as the governing BN. To incorporate the randomness of gene activity, a small perturbation probability *p* > 0 is assigned to each gene. This random perturbation allows all states of a PBN to communicate with each other, thereby resulting in an ergodic Markov chain with a SSD 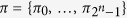. The long-term behavior of PBNs is characterized by this SSD. The simplest PBN is a Boolean network with perturbation *p* (BNp)^2^. A BNp inherits the attractor structure from the original BN without perturbation, the difference is that the random perturbation allows a BNp jump out of the BOA of an attractor and then evolves into the BOA of another attractor.

### One-bit perturbation

One-bit perturbation is the simplest function perturbation that flips the output value for a specific input of a Boolean function. As a function perturbation changes some of the state transitions, the BOA of some attractors may enlarge or shrink, some attractors may disappear, and some new attractors may appear. If gene *i* is regulated by *k*_*i*_ genes, then there are 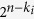 system states in which the input value for gene *i* is equal to a specific value 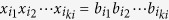 (*b*_*ij*_ ∈ {0, 1}). This means that a one-bit perturbation on this input value will lead to 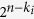 state-transition changes where the state of gene *i* is flipped. The detailed proof of this conclusion appears in ref. 9. Figure 1(C) shows the station-transition diagram after a one-bit perturbation of function 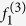 (the number “3” in parentheses indicates that the output of function *f*_1_ in the third row is flipped). We see that 2^5−3^ = 4 station-transition branches (marked by different colors) change their direction of evolution.

### Problem setting

Attractors represent the fixed points of dynamical systems and capture the system’s long-term behavior, so modifying attractor states may lead to severe side effects on the biological system in question. Therefore, to be prudent we should avoid one-bit perturbations that lead either to the disappearance of attractors or to the emergence of new attractors.

But it should be prudent to modify the fundamental landscaps.

In biology, cancer can be characterized by the imbalance between cellular states (attractors), such as proliferation and apoptosis (programmed cell death). The objective of intervention is to keep cells away from certain states (for example, metastatic cancerous phenotypes). In order to avoid unpredicted side effects on the biological system in question, it should be cautious to change the fundamental landscape of the state transitions, namely, the system’s attractor structure. In this paper, we restricted the one-bit perturbations that lead neither to the disappearance of attractors nor to the emergence of new attractors. Given the Boolean network *BN* with attractors *A*_1_, *A*_2_, …, *A*_*m*_, the first challenge is to identify such one-bit perturbations and avoid applying them.

The next challenge is to find the optimal intervention from the candidate one-bit perturbations in terms of maximizing the size of the BOA of expected attractors and minimizing that of unexpected attractors. As in previous studies, we classify the attractors into a desired attractor set *D* and an undesired attractor set *U* according to some target genes. The objective function to be maximized is


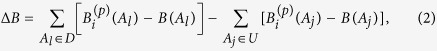


where *B*(*A*_*l*_) and 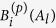 denote the size of the BOA for attractor *A*_*l*_ before and after perturbing 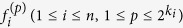, respectively. The size of the BOA of *A*_*l*_ is the sum of the number of transient states that eventually enter it and the number states |*A*_*l*_| included in it.

### Algorithm

To keep attractors unchanged, we must first identify the one-bit perturbations that may destroy the original attractors. Consider the singleton attractor *x*_1_*x*_2_*x*_3_*x*_4_*x*_5_ = 00100 for the BN in Fig. 1(A), the corresponding input value for gene *x*_1_ is *x*_5_*x*_2_*x*_4_ = 000. The one-bit perturbation 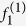 will lead the next state of 00100 being 10100, instead of being itself. Therefore, we should avoid considering this one-bit perturbation. By repeating this for other genes, we also find that the one-bit perturbations of 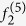, 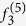, 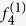, and 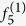 should also be avoided. By applying this process to all attractor states, we identify the one-bit perturbations that may destroy the attractors. In the truth table in Fig. 1(A), those one-bit perturbations have been marked by red color. However, some one-bit perturbations may lead to new attractors. Such perturbations are difficult to identify, so we leave this problem to be solved in the BOS-updating process. If we encounter a new attractor in the perturbed BN, we stop searching with the current one-bit perturbation (see Algorithm 3, lines 42–47). The pseudocode for identifying the one-bit perturbations that may affect attractor states is summarized in Algorithm 1.

**Algorithm 1: Mark the function bits which may destroy the original attractor states.**

**Input:** A Boolean network *G*(*V*, *F*) and *AS*;

**Output:** A function *F*’ with marked bits;

**1 begin**

**2**  *F*’ ← *F*;

**3  for** *i* ← 1 *to m*  // *m* is the number of attractors

**4**   *a* ← *AS*(*i*);

**5    for** *j* ← 1 *to n*

**6**     *r* ← *Input*_*RowNumber*(*a, j*);

     // extract the input row number of gene *j* in state *a*

**7**     *F*’ ← *mark*(*F*’*, j, r*);  // mark the *r*th output row of gene *j*

**8    end**

**9  end**

**10 return** *F*’

**11 end**

The second challenge is to identify the optimal structural intervention 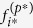 that maximizes the objective function Δ*B* in formula (2). Directly calculating the sizes *B*(*A*_*l*_) and 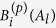
of the BOAs by an exhaustive search strategy is too time consuming for large BNs. Because the one-bit perturbation, 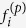 only changes 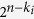 state transitions in the original state-transition diagram, we propose a framework to quickly determine the BOA size 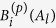 after the one-bit perturbation 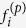.

Like the definition of BOA, we define the BOS of state *s* to be the transient states that can reach it. If state *s* is an attractor state, its BOS refers to those transient states which directly reach it, not including those transient states which reach it through other attractor states in the same cyclic attractor. For example in Fig. 1(B), a cyclic attractor includes two states 11010 and 11110. The BOS of attractor state 11110 only refers to those transient states: 01111, 01110, 00111, 00110 and 10010. The BOS size of state *s*, *BOS*(*s*), is the number of transient states plus 1. The BOA size of attractor *A*_*l*_ is then 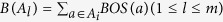.

Our motivation is that, if the BOS size *BOS*(*s*) for all states in the original network is known, then the BOA size *B*′(*A*_*l*_) for all attractors after a perturbation can be obtained by simply updating the state-transition diagram of the 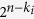 perturbed states.

To determine the BOS for all states and attractors *A*_1_, *A*_2_, …, *A*_*m*_ in a BN, we must travel from each state (00...00 to 11…11) to their corresponding attractors. Let 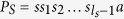 (*a* ∈ *A*_*l*_) denote the path from state *s* to its attractor *A*_*l*_. In the traversal process, the BOS size of the visited state *s*′ ∈ *s*_1_*s*_2_…*s*_*l*−1_*a* is incremented by 1 at each step. Two situations must be considered when arriving at state *a*: If *A*_*l*_ has previously been identified as an attractor, the traversal process should stop at state *a*. If *A*_*l*_ has not been identified as an attractor, the traversal process continues. When state *a* is revisited, the new attractor *A*_*l*_ can be identified. Finally, we should decrement by 1 the BOS size of the attractor states in *A*_*l*_ (apart from *a* itself). The pseudocode for determining the BOS size for all states and the attractors is summarized in Algorithm 2.

**Algorithm 2: Determine the basin of state (BOS) and the attractors set (AS).**

**Input:** A Boolean network *G*(*V*, *F*) and the evolution path *Next*;

**Output:** *BOS*;     // the *BOS* size of state *s*

*AS*;     // the states in all the currently identified attractors

**1 begin**

**2 begin**       // Initialization

**3**  *i* ← 0;     // the number of attractors

**4**  *set AS*(*i*) ← *ϕ*;

**5**  *BOS*(*s*) ← 0;

**6 end**

**7 for** *s* ← 1 *to* 2^*n*^ // calculate the *BOS*(*s*)

**8**   *SS* ← *ϕ*; // *SS* stores the states evolved from state *s*

**9**   *ss* ← *s*;

**10   while** (*ss* ∉ *AS and ss* ∉ *SS*)

**11**   {

**12**    *SS* ← *ss*;

**13**    *ss* ← *Next*(*ss*);

**14**    *BOS*(*ss*) ← *BOS*(*ss*) +1;

**15**   }

**16**  *BOS*(*s*) ← *BOS*(*s*) +1;

**17  if** (*ss* ∈ *AS*)   // enter an identified attractor**18**  *continue*;

**19  if** (*ss* ∈ *SS*)   // find a new attractor

**20**   {

**21**    *s0* ← *ss*;

**22**    *A* ← *ϕ*;  // the states in the *i*th identified attractor

**23    do**

**24**    {

**25**      *A* ← *ss*;

**26**      *BOS*(*ss*) ← *BOS*(*ss*) −1;

**27**      *ss* ← *Next*(*ss*);

**28**     } **while** (*ss* == *s0*);

**29**    *AS*(*i*) ← *A*;

**30**    *i* ← *i* + 1;  // increment the number of attractors

**31**   }

**32  end**

**33 return** *BOS and AS*

**34 end**

After identifying the BOS of all 2^*n*^ states and the attractors in a BN, the exhaustive strategy simply repeats Algorithm 2 to determine the size of the BOA for the perturbed networks. We propose herein an algorithm that uses the BOS size obtained in Algorithm 2 to determine the size of the BOA after a one-bit perturbation. Given a one-bit perturbation 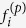, we assume *S*^*p*^ denotes the set of 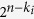 states whose transitions are changed. Our idea is that the ultimate BOS can be obtained by sequentially updating the path of each of the 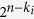 states. For each state *s* ∈ *S*^*p*^, the updating includes two processes: the SUB process and the ADD process. The SUB process updates the BOS size for all states in the current path *P*_*S*_, whereas the ADD process updates the BOS size for all states in the modified path 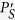.

**Figure 2:**
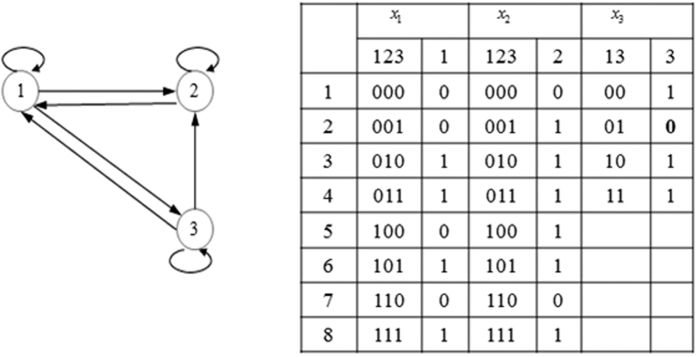
A simple BN consists of three genes and its truth table.

The updating process may produce some cycles. If the cycles are emerged cyclic attractors in the perturbed BN′, we should stop at once as the new attractor appears; otherwise, they are just temporary cycles formed in the updating process. The temporary cycles make the updating process more complicated. We now apply the network in Fig. 2 to illustrate the updating process in detail. The original BN contains eight states and one singleton attractor 111. The perturbation is implemented in the second bit of the function for the third gene. This intervention leads to a change in the state transitions of states 001 and 011.

**Figure 3:**
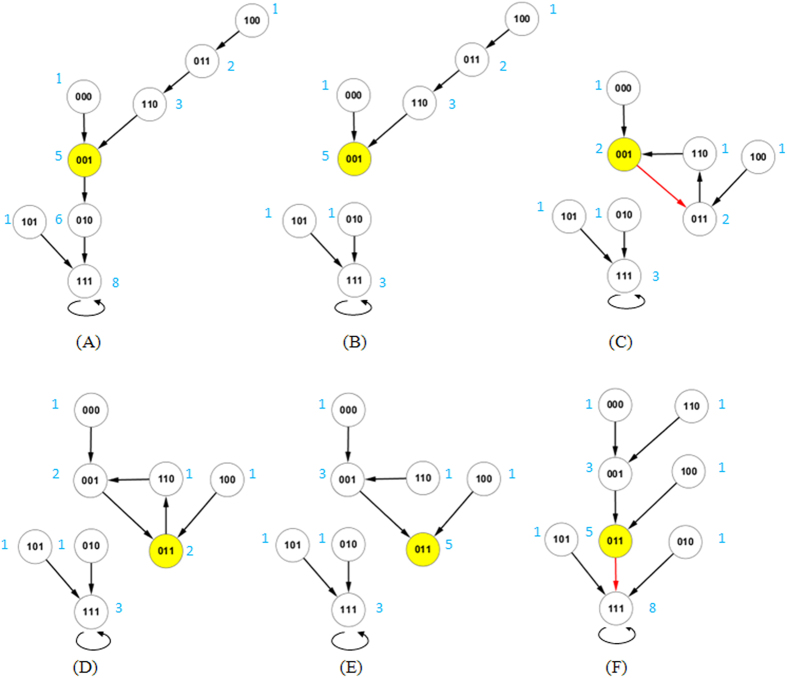
The SUB and ADD processes after the one-bit perturbation 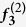.

For the SUB process, we first update the transition of state 001, which changes from state 111 to 011. Its current evolution path *P*_*S*_ is 001 → 010 → 111 [see Fig. 3(A)]. The BOS size of other states in *P*_*S*_ is simply updated by *BOS*(*s*′) − *BOS*(*s*); that is, the BOS sizes of 010 and 111 become 1 and 3, respectively [see Fig. 3(B)]. Next, we consider the SUB process for the transition of state 011. Its current path *P*_*S*_ is a temporary cycle 011 → 110 → 001 → 011 [see Fig. 3(D)]. In this case, its new transition destroys the cycle and leads to other states entering its BOS. Note that the BOS of state 110 does not change because the current path *P*_*S*_ is a cycle. The updating process for the BOS takes the following form: we first add *BOS*(110) to the BOS size of state 001, and then add the new *BOS*(001) to the BOS size of state 110 [see Fig. 3(E)].

For the ADD process, we also consider first the transition of state 001 to 011. This transition transforms the modified path 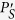 into a temporary cycle 001 → 011 → 110 → 001 [see Fig. 3(C)]. The consequence of the temporary cycle is that state 110 is no longer in the BOS of state 001, and state 011 is no longer in the BOS of state 110. Therefore, the BOS-updating process takes the following form: we first subtract *BOS*(110) from the BOS size of state 001, and then we subtract *BOS*(011) from the BOS size of state 110. Next, we consider the ADD process for the transition of state 011. Its modified path 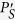 is 011 → 111, which directly enters an attractor. The BOS-updating process simply adds *BOS*(011) to other states in 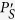 [see Fig. 3(F)]. The pseudocode to determine the updated-BOS size for all states is summarized in Algorithm 3.

**Algorithm 3: Determine the basin of state (BOS) under a one-bit perturbation.**

**Input:** A Boolean network *G*(*V*, *F*), *BOS*, *AS, Next* and the perturbation bit 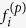;

**Output:** *BOS*’;  // the *BOS* size of state *s* after intervention**1 begin**

**2  begin**

**3**  *BOS*’ ← *BOS*;  // stores the *BOS* size of state *s* in the process of evolution

**4**  *Next*’ ← *Next*;  // stores the current path in the process of evolution

**5**   *AS*’ ← *AS*;

**6**  *S*^*p*^ ← *Affectedstates*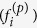; // *S*^*p*^ is the 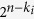 affected states after perturbation

**7   end**

**8   for** *l* ← 1 *to* 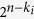


**9**    *s* ← *S*^*p*^(*l*);

**10**   *ss* ← *Next*’(*s*);

**// the SUB process**

**11   if** *s* ∉ *AS*′   **//** *s* does not belong to *AS*’

**12    while** (*ss* ∉ *AS*′)

**13**    {

**14**     *BOS*’(*ss*) ← *BOS*’(*ss*) – *BOS*’(*s*); // SUB the *BOS*(*s*) from its next state in                   the current state-transition diagram

**15**    *ss* ← *Next*’(*ss*);

**16**   }

**17**   *BOS*’(*ss*) ← *BOS*’(*ss*) − *BOS*’(*s*);

**18   else      //** while *s* is an attractor in an attractor cycle

**19**   *SS* ← *ss*;

**20   while** (*ss* ≠ *s*)

**21**   {

**22**    *BOS*’(*Next*’(*ss*)) ← *BOS*’(*Next*’(*ss*)) + *BOS*’(*ss*);

**23**     *ss* ← *Next*’(*ss*);

**24**     *SS* ← *SS* U *ss*;

**25**   }

**26**   *AS*’ ← *AS*’ **-** *SS*;  // decrease the vanished attractor cycle from *AS*’

**27  end**

**// the ADD process**

**28**   *ss* ← *Nextstate*(*s*);

**29**   *Next*’(*ss*) ← *ss*;

**30**   *SS* ← *ϕ*; *p* ← 0;

**31   while** (*ss* ≠ *s and ss* ∉ *AS*′)

**32**   *p* ← *p* + 1;

**33**    *SS*(*p*) ← *ss*;    // *SS* stores the evolution path after intervention

**34**    *ss* ← *Next*’(*ss*);

**35   end**

**36**  *p* ← *p* + 1;

**37**   *SS*(*p*) ← *ss*;

**38   if** *ss* ∈ *AS*′

**39**    *BOS*’(*SS*(*1*:*p*)) ← *BOS*’(*SS*(*1*:*p*)) + *BOS*’(*s*);

**40  else**       //it forms a new attractor or attractor cycle

**41**  {// determine whether the new attractor cycle should be a break

**42    for** *l* ← 1 *to p* − 1

**43**    *ss*_*m*(*l*) ← *Next state* (*SS*(*l*));

**44**    *ss*_*n*(*l*) ← *SS*(*l* + 1);

**45    end**

**46    if** *p* == 1 *or all* (*ss*_*m* == *ss*_*n*)

**47     break;**

**48    else**

**49    for** *ii* ← *p to* 2

**50**     *BOS*’(*SS*(*ii*)) ← *BOS*’(*SS*(*ii*)) - *BOS*’(*SS*(*ii*-1));

**51**     *ii* ← *ii* − 1;

**52    end**

**53**     *AS*’ ← *AS*’ U *SS*;  // increase the new attractor cycle to *AS*’

**54    end**

**55**   }

**56 end**

**57 return** *BOS*’;

**58 end**

**Figure 4:**
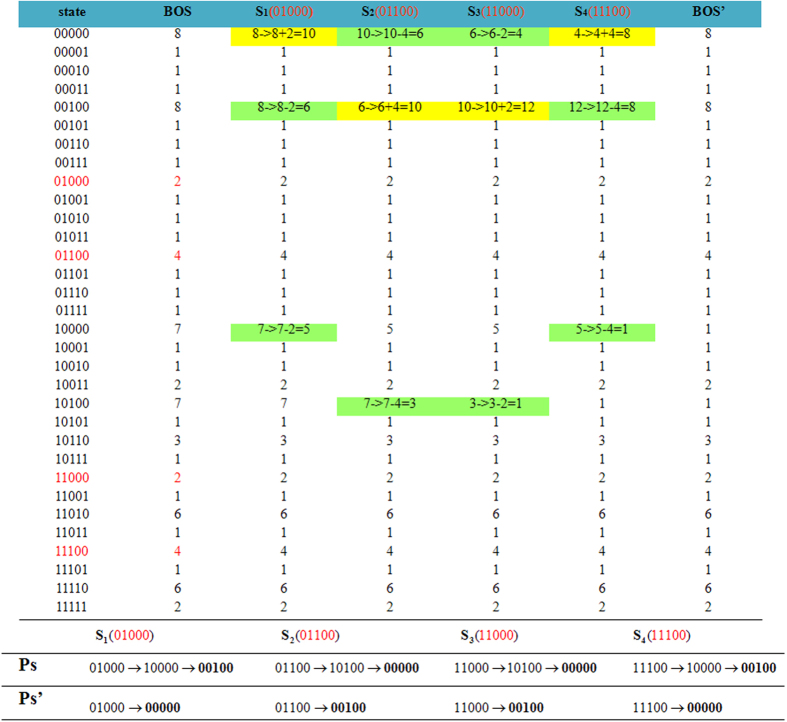
Evolution of the BOS size for BN in Fig. 1 under the one-bit perturbation 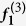.

Figure 4 presents the process for updating the BOS size for the BN in Fig. 1 under a one-bit perturbation 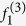. This perturbation affects the transitions of four states: 01000, 01100, 11000, and 11100. The first column lists the 32 states, and the second column is the BOS of each state in the original BN. The following four columns present the process for updating the BOS size according to the order of the four states. The updating of the current path *P*_*S*_ is highlighted in green and that of the modified path 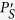 is highlighted in yellow. The last column is the resulting BOS size for all states. This table shows that the updating process visits very few parts of the whole state space. Therefore, it is less time consuming than the exhaustive strategy.

Based on algorithms 2 and 3, the workflow of our BOS-based algorithm to identify the optimal one-bit perturbation includes the following main steps:

**Step 1:** Apply Algorithm 2 to build the BOS size of all states in the original BN and identify its attractors.

**Step 2:** Apply Algorithm 1 to identify the one-bit perturbations that may destroy the original attractors.

**Step 3:** For a potential one-bit perturbation 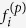, apply Algorithm 3 to obtain the updated BOS size of all states.

**Step 4:** Calculate Δ*B* for each feasible one-bit perturbation.

**Step 5:** Repeat Step 3 for other potential perturbations and find the perturbation that gives the maximal Δ*B*.

## Results and Discussion

In this section, we compare the SSD algorithm, the exhaustive algorithm, and the proposed BOS-based algorithm both in simulated networks and in two biological networks. All calculations use the PBN Toolbox (http://code.google.com/p/pbn-matlab-toolbox/), which calculates the SSD. We implemented both the exhaustive and the BOS-based algorithms with this toolbox. All numerical experiments were done on a windows computer with two Core 2.4 GHz processors and 32 GB of physical memory.

### Simulation on synthetic networks

**Figure 5:**
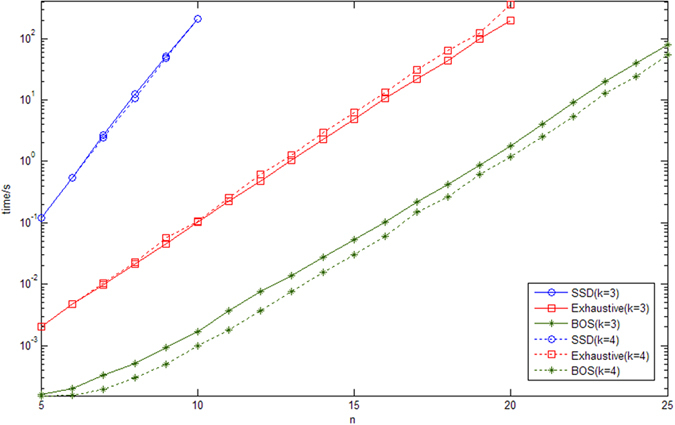
Average time to calculate the size of the BOA or the SSD for a one-bit perturbation. Solid lines are for *K* = 3 and dashed lines are for *K* = 4.

To compare the performance of the three algorithms on synthetic networks, we randomly generate 20 BNs for each number *n* of genes (*n* = 5, …, 25) and the average indegree *K* (*K* = 3, 4). The perturbation probability of each gene used to calculate the SSD is *p* = 0.001. Figure 5 shows the average time of the three algorithms for a one-bit perturbation. The solid lines are for *K* = 3 and the dashed lines are for *K* = 4.

First, the BOS-based algorithm performs best while the SSD-based algorithm performs worst. In particular, the time needed to calculate the SSD increases with the number *n* of genes more quickly than the time required to directly calculate the BOA size of all attractors. This is apparent by observing the slope of the lines in Fig. 5.

Second, the ratio of the average computing time for the exhaustive algorithm to that for the BOS-based algorithm is almost constant. This result is reasonable because, if the average indegree is *K*, then the BOS-based algorithm only needs to update the evolution path of the 2^*n*−*K*^ states whose transitions have been changed, whereas the exhaustive algorithm has to do the same for all 2^*n*^ states.

Third, the average indegree *K* does not influence the performance of the SSD algorithm. However, it affects the performance of both the exhaustive and the BOS-based algorithms. Concerning the exhaustive algorithm, the average computing time for *K* = 4 is slightly longer than that for *K* = 3. One major reason for this result is that the average length from a state to its attractor for BNs with *K* = 4 is longer than for BNs with *K* = 3. Therefore, it takes longer to determine the size of the BOA for *K* = 4 than for *K* = 3^13^. Concerning the BOS-based algorithm, the effect of the average indegree is completely the opposite because 2^*n*−*K*^ states are affected by a one-bit perturbation. Therefore, given a specific number *n* of genes, the BOS-based algorithm needs to update fewer states for BNs with *K* = 4 than for BNs with *K* = 3.

Apart from the time issue, the space complexity of the SSD is *O*(2^*n*^ × 2^*n*^), whereas that of the other two algorithms is only *O*(2^*n*^). Therefore, the proposed BOS-based algorithm can be applied to larger BNs than the other two algorithms. Figure 5 shows that the proposed BOS-based algorithm takes about 80 seconds for BNs with 25 genes whereas the exhaustive method takes about 300 seconds even for BNs with 20 genes.

### Biological networks

In this section, we apply the three algorithms to two biological networks: the metastatic melanoma network^14^ and the T-helper network^15^. The metastatic melanoma network contains seven key genes: WNT5A, pirin, S100P, RET1, MART1, HADHB, and STC2, which are labeled *x*_1_, …, *x*_7_. It has four singleton attractors: 0101111, 0110110, 0111110, and 1000001. This network was used in previous works to illustrate the effectiveness of various intervention strategies. Increasing the levels of the Wnt5a protein through a melanoma cell line is believed to alter the competence of the cell. Therefore, attractor 1000001 is undesirable, whereas the others are desirable. The T-helper network has 23 key genes: GATA3, IFN-ß, IFN-ßR, IFN-γ, IFN-γR, IL-10, IL-10R, IL-12, IL-12R, IL-18, IL-18R, IL-4, IL-4R, IRAK, JAKI, NFAT, SOCS1, STAT1, STAT3, STAT4, STAT6, T-bet, and TCR, which are labeled *x*_1_, …, *x*_23_. The average indegree of this network is *K* = 1.7. This network has 33 single attractors, which can be classified as 16 undesirable attractors and 17 desirable attractors: *x*_1_ = 0 and *x*_1_ = 1, respectively.

**Figure 6:**
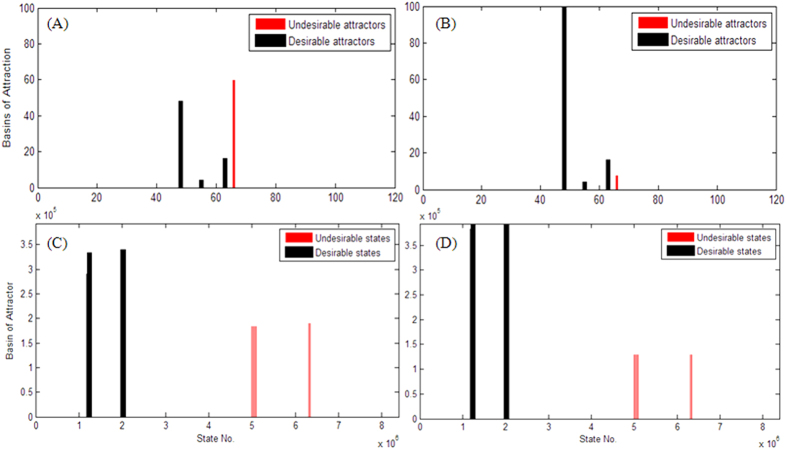
Distributions of size of BOAs of desired and undesired attractors for two biological networks: the metastatic-melanoma network and T-helper network. Panels (**A**,**B**) show the distributions before and after the optimal one-bit perturbation for the metastatic-melanoma network. Panels (**C**,**D**) show the distributions before and after the optimal one-bit perturbation for the T-helper network.

Figure 6 presents the size of the BOA of the two networks before and after the optimal one-bit perturbation identified by the BOS-based algorithm. Red bars denote the size of the BOA for the undesirable attractors and black bars denote the size of the BOA for desirable attractors. First, the identified optimal one-bit perturbations retain the attractors of the networks. Second, the optimal one-bit perturbation obviously reduces the size of the BOA of the undesirable attractors. Concerning the metastatic melanoma network, the optimal one-bit perturbation identified by the BOS-based algorithm is 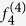, which can both retain the attractors of the system and maximally reduce the size of the BOA of undesirable attractors. This result is consistent with previous studies in ref. 10.

To identify the optimal one-bit perturbation, Table 1 presents the computing time for the three algorithms. The time for the SSD-based algorithm is not presented because it is impossible to deal the state-transition matrix of size 2^23^ × 2^23^ with currently available computers. The exhaustive algorithm takes about 20 hours to identify the optimal one-bit perturbations, whereas the proposed BOS-based algorithm takes only about three minutes.

## Conclusions

From the point of view of biology, structural intervention has the potential to permanently alter the dynamic behavior of gene regulatory networks, and then push the system so that it evolves in a desirable direction. Although previous studies applying matrix perturbation theory ensure that the perturbation obtained is the optimal one-bit perturbation that can maximally shift the probabilistic mass of the SSD toward desirable states, its application is limited to networks with only about 15 genes.

Although Xiao *et al.* proposed to directly observe the changes in the size of the BOA before and after a function perturbation, they did not give a systematic computational algorithm that can efficiently solve this problem. In this paper, we propose an algorithm that efficiently determines the size of the BOA after a one-bit perturbation. To avoid unexpected side effects, we require that the optimal one-bit perturbation maintain the same network attractors. Our motivation is that, if we know the BOS size of all states, then the modified size of the BOA of all attractors after a one-bit perturbation of 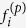 can be obtained by updating only the evolution path of the 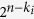 states whose transition have been altered. Results obtained from both synthetic networks and two real biological networks show that the proposed BOS-based algorithm is more efficient than both the exhaustive algorithm and the SSD-based algorithm. According to the results of the numerical experiments, the proposed BOS-based algorithm can be applied to networks with 25 genes. Finally, note that the time complexity of the proposed BOS-based algorithm is still exponential. To solve the optimal-intervention problem for larger BNs, we must study other methods to determine the size of the BOA of perturbed networks quickly.

## Additional Information

**How to cite this article**: Hu, M. *et al.* An efficient algorithm to identify the optimal one-bit perturbation based on the basin-of-state size of Boolean networks. *Sci. Rep.* **6**, 26247; doi: 10.1038/srep26247 (2016).

## Figures and Tables

**Table 1:** Performance of the three algorithms for two real biological networks.

Benchmark	n	K	Time (s)		
			BOS-based	Exhaustive	SSD
Wnt5a	7	2.6	0.004	0.55	51.5
T-helper	23	1.7	175.4	73636	

## References

[b1] Shmulevich I., Dougherty E. R., Kim S. & Zhang W. Probabilistic Boolean Networks: A Rule-Based Uncertainty Model for Gene Regulatory Networks. Bioinformatics 18, 261–274, 10.1093/bioinformatics/18.2.261 (2002). 11847074

[b2] Shmulevich I. & Dougherty E. R. Genomic Signal Processing (Princeton Series in Applied Mathematics). (Princeton University Press, 2007).

[b3] Ivanov I. & Dougherty E. R. Modeling Genetic Regulatory Networks: Continuous or Discrete? Journal of Biological Systems 14, 219–229, 10.1142/S0218339006001763 (2006).

[b4] Shmulevich I., Dougherty E. R. & Wei Z. From Boolean to Probabilistic Boolean Networks as Models of Genetic Regulatory Networks. Proceedings of the IEEE 90, 1778–1792, 10.1109/JPROC.2002.804686 (2002).

[b5] Shmulevich I., Dougherty E. R. & Zhang W. Gene Perturbation and Intervention in Probabilistic Boolean Networks. Bioinformatics 18, 1319–1331, 10.1093/bioinformatics/18.10.1319 (2002). 12376376

[b6] Qian X., Ivanov I., Ghaffari N. & Dougherty E. R. Intervention in Gene Regulatory Networks Via Greedy Control Policies Based on Long-Run Behavior. BMC Systems Biology 3, 1–16, 10.1186/1752-0509-3-61 (2009). 19527511 PMC2728102

[b7] Yousefi M. R. & Dougherty E. R. Intervention in Gene Regulatory Networks with Maximal Phenotype Alteration. Bioinformatics 29, 1758–1767, 10.1093/bioinformatics/btt242 (2013). 23630177

[b8] Shmulevich I., Dougherty E. R. & Zhang W. Control of Stationary Behavior in Probabilistic Boolean Networks by Means of Structural Intervention. Journal of Biological Systems 10, 431–445, 10.1142/S0218339002000706 (2002).

[b9] Xiao Y. & Dougherty E. R. The Impact of Function Perturbations in Boolean Networks. Bioinformatics 23, 1265–1273, 10.1093/bioinformatics/btm093 (2007). 17379691

[b10] Qian X. & Dougherty E. R. Effect of Function Perturbation on the Steady-State Distribution of Genetic Regulatory Networks: Optimal Structural Intervention. IEEE Transactions on Signal Processing 56, 4966–4976, 10.1109/TSP.2008.928089 (2008).

[b11] Qian X., Yoon B. J. & Dougherty E. R. Structural Intervention of Gene Regulatory Networks by General Rank-K Matrix Perturbation. *In Proceedings of 2012 IEEE International Conference on Acoustics, Speech and Signal Processing*(*ICASSP*), Kyoto, Japan. Washington DC, USA: IEEE Computer Society, 10.1109/ICASSP.2012.6287987 (2012, March 25–30).

[b12] Bouaynaya N., Shterenberg R. & Schonfeld D. Inverse Perturbation for Optimal Intervention in Gene Regulatory Networks. Bioinformatics 27, 103–110, 10.1093/bioinformatics/btq605 (2011). 21062762 PMC3008638

[b13] Zheng Q., Shen L., Shang X. & Liu W. Detecting Small Attractors of Large Boolean Networks by Function-Reduction-Based Strategy. IET Systems Biology 10, 49–56, 10.1049/iet-syb.2015.0027 (2016). 26997659 PMC8687213

[b14] Weeraratna A. T. *et al.* Wnt5a Signaling Directly Affects Cell Motility and Invasion of Metastatic Melanoma. Cancer Cell 1, 279–288, 10.1016/S1535-6108(02)00045-4 (2002). 12086864

[b15] Mendoza L. & Xenarios I. A Method for the Generation of Standardized Qualitative Dynamical Systems of Regulatory Networks. Theoretical Biology and Medical Modelling 3, 1–18, 10.1186/1742-4682-3-13 (2006). 16542429 PMC1440308

